# “Viral Suppression Among Rural and Urban People Living With HIV in Wyoming”

**DOI:** 10.1155/arat/7353282

**Published:** 2026-02-23

**Authors:** Sarah Hendricks, Anh Huynh, Daniel T. Burns, Shawna Pena, Paul Johnson

**Affiliations:** ^1^ Communicable Disease Unit, Wyoming Department of Health, 122 W. 24th St 3rd Floor West, Cheyenne, 82002, Wyoming, USA; ^2^ University of Washington School of Medicine, 1959 NE Pacific St, Seattle, 98195, Washington, USA, uwmedicine.org; ^3^ Department of Mathematics and Statistics, University of Wyoming, Dept. 3036 1000 E. University Ave., Laramie, 82071, Wyoming, USA, uwyo.edu; ^4^ Division of Healthcare Financing, Wyoming Department of Health, 122 W. 24th St 4th Floor West, Cheyenne, 82002, Wyoming, USA; ^5^ Department of Otolaryngology-Head and Neck Surgery, University of Washington, 1959 NE Pacific St, Seattle, 98195, Washington, USA, washington.edu

**Keywords:** HIV, rural, Ryan White, viral suppression

## Abstract

**Purpose:**

To determine whether sociodemographic factors, including residence in rural/frontier versus urban settings, are associated with differences in HIV viral suppression rates among people living with HIV in Wyoming with one or more reported viral load results in 2023.

**Methods:**

Wyoming Department of Health databases were used to identify people living with HIV in Wyoming who had a viral load reported between January 1, 2023, and December 27, 2023. Demographic information was obtained, including county of residence, sex, age, insurance status, and Ryan White HIV and AIDS Program participation. Multiple logistic regression models were developed to conduct a cross‐sectional examination of the effect of geographic location on the viral suppression status.

**Findings:**

A total of 272 people were identified as having one or more viral load results in 2023 and were included in the study. A total of 248 study participants were virally suppressed. There was no significant association between rural/frontier and urban county of residence and the odds of being virally suppressed (OR = 1.066, 95% CI: [0.447–2.617], and *p* = 0.886). There was a significant association between age and viral suppression status, with the odds of being virally suppressed increasing as age increases (OR = 1.046, 95% CI: [1.012–1.082], and *p* = 0.008).

**Conclusions:**

There was no statistically significant association in HIV viral suppression between rural/frontier and urban people living with HIV in Wyoming who had a viral load result in 2023. There was a statistically significant association between age and odds of viral suppression, with the odds of viral suppression increasing as age increases.

## 1. Introduction

One of the primary goals of HIV treatment is to achieve viral suppression. While a viral load between 200 and 1000 copies/mL is considered suppressed by the World Health Organization, the Centers for Disease Control (CDC) uses the lower threshold of a viral load below 200 copies/mL as a benchmark metric for the United States (US) [1]. At this level, there is a significantly reduced risk of disease progression and transmission to others [2]. As such, achieving viral suppression is a cornerstone to managing the HIV epidemic. Efforts to screen, test for, and treat HIV are critical to meeting this goal. Federal programs such as the Ryan White HIV and AIDS Program (RWHAP) can assist people living with HIV (PLWH) in achieving viral suppression by providing access to holistic care and services. Services offered by the program include coverage of HIV antiretroviral therapy (ART) medications, HIV‐related healthcare, insurance premiums, and case management [3]. Current literature shows this program is particularly effective for achieving viral suppression in rural settings [4].

While the importance of viral suppression is well established and programs, such as RWHAP, that provide medical and support services are available, current literature shows viral suppression rates are lower in rural populations compared with urban populations [5–8]. The literature also shows that low suppression rates may be correlated with multiple sociodemographic factors, including sex, race, poverty level, and insurance status [9–12]. As of 2022, Black and Hispanic/Latino persons accounted for 70% of all HIV infections, and the lowest viral suppression rates are among women and people who are Black [11]. Lower viral suppression rates have also been shown among PLWH with Medicaid coverage or no insurance compared with other coverage types [12].

With these factors in mind, we designed a cross‐sectional study to analyze viral suppression rates of PLWH residing in rural/frontier compared with urban settings of Wyoming based on viral load results reported to the Wyoming Department of Health. We also wanted to determine if any associations existed between viral suppression rates and specific sociodemographic factors, including age, race, sex, insurance status, and enrollment in Wyoming’s RWHAP.

Wyoming is the least populated state in the US with the lowest prevalence of PLWH. US census data from 2020 report 576,851 people reside in the state, and in 2023, 394 Wyoming residents older than age 13 were reported to the National HIV Surveillance System as living with HIV [13, 14]. Of those 394 residents, 206 (52%) were enrolled in the Wyoming Ryan White program. Wyoming has 23 counties and as of 2024, two are considered urban, four rural, and seventeen frontier by the Health Resources and Services Administration (HRSA) [15]. HRSA defines rural as at least 400 square miles in area with a population density of 35 or fewer people per square mile, while frontier is defined as fewer than six people per square mile [15, 16]. These seventeen frontier counties accounted for 46% of the population’s residences in 2020 [15].

## 2. Methods

### 2.1. Data

To create our cross‐sectional study, we used the Wyoming Department of Health’s (WDH) Enhanced HIV/AIDS Reporting System (eHARS) to identify all people living with HIV in Wyoming who had a viral load reported between January 1, 2023, and December 27, 2023. This database reports viral load results based on where the patient primarily resides, not where they seek care. As such, this we did not need to be concerned about PLWH who seek care in bordering states to due to stigma or confidentiality concerns. PLWH who did not have a viral load drawn were not included in the eHARS dataset, and, therefore, the missing data points could not be imputed or estimated, and they were not included in the study. In addition to viral load results, patient‐level sociodemographic information was obtained, including ZIP code of residence, sex, age, and insurance status. These data were cross‐referenced with Wyoming’s RWHAP CAREWare data system using persons’ first name, last name, and date of birth to determine the number of PLWH in Wyoming who had viral loads drawn and were also enrolled in the state’s RWHAP during the study period. ART use and adherence were not assessed in our study. All data collected from these datasets were deidentified by Wyoming RWHAP staff and an application for this research was submitted to the Wyoming Department of Health Institutional Review Board (IRB), prior to being shared with coauthors for analysis to maintain ethical integrity.

### 2.2. Variables

Viral load, the outcome of interest, was dichotomized into two groups: virally suppressed (viral load ≤ 200 copies/mL) or not virally suppressed (viral load > 200 copies/mL) [2]. For individuals with multiple viral load results reported in the study period, the most recent result was used for this study.

Variables investigated against viral suppression included the following:

• Rural, frontier, or urban residence
• Age
• Race
• Sex
• Insurance status
• RWHAP enrollment

Frontier, rural, and urban locations at the county level were determined using the HRSA definition [14]. Race was categorized as “White” or “non‐White.” Sex assigned at birth was categorized as “Female” or “Male.” Insurance status was categorized as “no” (uninsured), “yes” (insured), and “unknown.” Age was studied as a continuous variable. Individuals who had insurance at any point in the study period were categorized as “yes” for this study. RWHAP enrollment was categorized as “yes” (enrolled) or “no” (not enrolled). Individuals enrolled in the program at any point in the study period were categorized as “yes” for this study. Baseline characteristics by the suppression status are shown in Table 1.

**TABLE 1:** Description of study sample by variables and HIV viral suppression status.

Sociodemographic factor (*n* = 272)	Categories for factor	Virally suppressed	
		No (*n* = 24)	Yes (*n* = 248)
Age (years)	Mean (SD)	44.3 (11.0)	52.0 (13.1)
Age (years)	Range	25–70	17–82
RWHAP status	No	5 (20.8)	70 (28.2)
	Yes	19 (79.2)	178 (71.8)

Insurance status	No	5 (20.8)	33 (13.3)
	Unknown	3 (12.5)	42 (16.9)
	Yes	16 (66.7)	173 (69.8)

Biological sex	Female	4 (16.7)	42 (16.9)
	Male	20 (83.3)	206 (83.1)

Race	Non‐white	5 (20.8)	34 (13.7)
	White	19 (79.2)	214 (86.3)

Location	Frontier	14 (58.3)	138 (55.6)
	Urban	10 (41.7)	110 (44.4)

*Note:* Age is shown as mean (SD) while all other characteristics are counts (%). Note also that rural and frontier classifications have been combined as Frontier for these data.

Abbreviation: RWHAP = Ryan White HIV/AIDS Program.

### 2.3. Data Analysis

The first model tried (not shown) contained the original three categories for geographic location: rural, urban, and frontier (RUF). After preliminary examination of the data, frontier and rural locations were grouped into the “frontier” group, as there were no differences in viral suppression.

Multiple logistic regression models were developed to examine the effect of geographic location on the viral suppression status. Individual socioeconomic factors were initially included as potential confounders. To find the most parsimonious model to explain the association while still accounting for confounders, backwards stepwise selection was performed, where the model with the lowest Akaike Information Criterion (AIC) value was chosen (see Appendix A for the model selection process). This model selection process selected the variable age, and geographic location was added back in as this was the variable of interest. The RWHAP participation status was then added back in as it was deemed a confounder. A *p* value of < 0.05 was considered statistically significant, and a change to the coefficient associated with geographic location of ≥ 10% after removing potential confounders was used to determine confounding. Analyses were performed using *R* Statistical Software v4.3.2 (R Foundation for Statistical Computing).

## 3. Results

Our study cohort included 272 PLWH with a viral load result reported to the Wyoming Department of Health within the study period. Of the 272 PLWH in the study cohort, 248 (91.2%) were virally suppressed. As shown in Table 1, of those who were virally suppressed, 178 (71.8%) participated in the Wyoming RWHAP, 206 (83.1%) were male, 214 (86.3%) were white, 138 (55.6%) lived in a rural/frontier area, and 173 (69.8%) had insurance. The average age was 52 years. Of those who were not virally suppressed, 19 (79.2%) participated in the Wyoming RWHAP, 20 (83.3%) were male, 19 (79.2%) were white, 14 (58.3%) lived in a rural/frontier area, and 16 (66.7%) had insurance. The average age was 44.3 years.

There was no statistically significant difference between rural and frontier geographic location nor were there any meaningful differences in the distributions of the other variables when grouped based on location (Supporting Table A4). The lack of meaningful difference in viral suppression between rural and frontier residence, combined with Wyoming’s small study cohort, led to the two groups being merged into one larger group for comparison to the state’s two urban areas.

The findings for the multivariate logistic regression are shown in Table 2. There was no significant association between the rurality of residence and the odds of being virally suppressed (odds ratio = 1.066, 95% CI: [0.447–2.617], and *p* = 0.886). There was a significant association between age and viral suppression status, with the odds of being virally suppressed increasing as age increases (odds ratio = 1.046, 95% CI: [1.012–1.082], and *p* = 0.008). There were no significant associations between the other factors studied (race, sex, insurance status, and RWHAP enrollment) and the odds of being virally suppressed.

**TABLE 2:** Logistic regression results showing odds ratios for viral suppression by selected factors.

	Estimate	SE	Odds ratio (95% CI)	Statistic	*p* value
(Intercept)	0.448	0.893	1.565 (0.283–9.692)	0.501	0.616
RWHAP	−0.386	0.534	0.68 (0.214–1.816)	−0.723	0.470
Age	0.045	0.017	1.046 (1.012–1.082)	2.633	0.008^∗^
Location	0.064	0.446	1.066 (0.447–2.617)	0.144	0.886

*Note:* Location variable refers to the geographic indicator where frontier is the reference group compared with the urban group. RWHAP refers to participation in the Ryan White Program. Note also that rural and frontier classifications have been combined as “frontier” for these data.

Abbreviation: RWHAP = Ryan White HIV/AIDS Program.

^∗^ *p* < 0.05.

## 4. Discussion

We found no statistically significant association between rural/frontier versus urban county of residence and the odds of viral suppression among our study cohort of PLWH in Wyoming. We also found no statistically significant association between the odds of viral suppression and the other factors studied (race, sex at birth, and insurance status). The only significant statistical association between viral suppression and the factors analyzed was with age, in which the odds of being virally suppressed increased with aging. While this association is statistically significant, it is important to note our cross‐sectional analysis cannot establish causality and prove that being older causes viral suppression. Although we found no statistically significant associations in viral suppression rates between rural/frontier and urban populations or other sociodemographic factors besides age, some variables and factors could explain our results.

First, the demographic profile of our study cohort differs from the national representation of PLWH. Our study cohort was 80% White, male, and above the age of 50. These factors have all been correlated with higher viral suppression rates [11, 17]. Specifically, adults over 50 have a reduced risk of nonadherence to ART, a significant determinant in achieving and maintaining viral suppression [17].

Second, our results showed that of the 197 study participants who were enrolled in the RWHAP, 90% were virally suppressed. While this was comparable with the national RWHAP viral suppression average (90.6%), the large percentage (72%) of our study cohort participating in Wyoming’s RWHAP positively contrasts with the approximately 50% of PLWH enrolled in RWHAP nationally [17]. Wyoming’s RWHAP model leverages local public health offices to provide access to case management services and HIV treatment in all of the state’s 23 counties. This model mitigates barriers to care and could explain why there was not the expected rural vs. urban disparity in viral suppression seen in other studies [5–10].

Lastly, an important strength of our study was our robust data systems, which allowed us to perform a cross‐sectional study on viral suppression for the majority of PLWH in Wyoming. In addition to the large number of PLWH in our study cohort enrolled in the RWHAP, we were able to assess viral suppression of non‐RWHAP PLWH in our study cohort to discover 93% (70) were virally suppressed compared with the national average of 65% [18].

### 4.1. Limitations

Wyoming has the lowest prevalence of HIV in the country, which contributed to our small study cohort that also had a very homogenous population [11]. While our small study cohort numbers necessitated collapsing race into White vs. non‐White, we acknowledge this could hide heterogeneity and not be in alignment with national surveillance standards. These unique characteristics of our study cohort limit our findings generalizability to more diverse rural populations.

Our study also made no adjustments for ART regimen initiation/adherence or time since diagnosis which may influence suppression levels. Additionally, PLWH who did not have at least one viral load result reported during the study period (122 individuals, 30% of total prevalence in 2023) were not included. It is possible that the missing data points from these individuals could have influence our results.

Lastly, the limitations of stepwise regression, primarily the possibility of overfitting and biased parameter estimation, apply to this study. The small number of individuals who were unsuppressed limits the power to detect differences and associations, especially in a multivariable regression; adding too many variables to model with such a small group size risks overfitting, thus further necessitating parsimony.

## 5. Conclusion

The results of this study show there was not a statistically significant association in viral suppression within our study cohort of PLWH living in rural/frontier and urban settings in Wyoming in 2023. There were also no statistically significant associations in viral suppression for other analyzed sociodemographic factors within our study cohort except for age. The study did show a statistically significant positive correlation between viral suppression and older age, which aligns with current literature. It also showed that Wyoming, a predominantly rural state, had high viral suppression rates within our study cohort, which contradicts current literature for other rural cohorts with different population compositions [5–10]. While the characteristics of our study cohort limits the generalizability of our findings, the lack of disparities observed in this study could be contributed to high RWHAP coverage in Wyoming and the uniqueness of the cohort’s composition, rather than reflecting an absence of rural disadvantage.

Key priorities moving forward include achieving a better understanding of the underlying factors contributing to Wyoming’s high viral suppression rates, examining additional data points, such as PLWH who did not have viral load results, using alternative rural definitions for data analysis, and identifying the clinical and programmatic solutions that best support viral suppression for PLWH in rural settings. Additional research examining the composition of HIV‐positive populations in rural areas across the US are needed to establish generalizable data and develop evidence‐based strategies.

## Funding

No funding was received for this manuscript.

## Conflicts of Interest

The authors declare no conflicts of interest.

## Supporting Information

The Akaike information criterion (AIC) is a popular, well‐known model selection tool, and the model with the lowest AIC value is considered best [19]. The built‐in step function in *R* was used to select the best model containing some combination of the variables. The function works by first being given a model and then iteratively removing one variable at a time to obtain the best (lowest) AIC value. The complete model was utilized, containing the six variables mentioned in the Data section and shown in Supplemental Table A4. The step function measures the AIC value obtained if each variable were removed while leaving the others in the model. As an example, the first two iterations are shown in Supplemental Tables A1, A2, and A3, where “none” refers to not removing any variable. Supplemental Table A1, the first step, shows removing the insured variable from the full model results in the lowest AIC value. Supplemental Table A2 shows the next step that considers the newly updated model (the five remaining variables with the insured variable removed) and selects a variable to remove to obtain a lower AIC value. In this case, the model with the lowest AIC value was reached at the fifth iteration, as seen in Supplemental Table A3.

## Supporting information


**Supporting Information** Additional supporting information can be found online in the Supporting Information section.

## Data Availability

Data that support the findings of this study are available on request from the corresponding author. The data are not publicly available due to privacy or ethical restrictions.
