# Supplementary material for: “Viral Suppression Among Rural and Urban People Living With HIV in Wyoming”
Source: AIDS Res Treat. 2026 Feb 23;2026:7353282. doi: 10.1155/arat/7353282 (PMC12927948; doi:10.1155/arat/7353282)
Supplement: Supplementary file 1 — Supporting Information Additional supporting information can be found online in the Supporting Information section. [file ARAT-2026-7353282-s001.docx]

**Supplemental Table A1: First Step Function Measure of AIC Value**

| **First Step (AIC = 171.17)**  suppression ~ insured + RUF + sex + RWHAP + race + age | |
| --- | --- |
| Variable Removed | AIC |
| Insured | 167.3 |
| RUF | 167.97 |
| Sex | 169.19 |
| RWHAP | 169.23 |
| Race | 169.34 |
| None | 171.17 |
| Age | 175.36 |

The first step in AIC-based model selection. Removing the insured variable from the full model results in the lowest AIC value.

**Supplemental Table A2: Second Step Function Measure of AIC Value**

| **Second step (AIC = 167.3)**  suppression ~ RUF + sex + RWHAP + race + age | |
| --- | --- |
| Variable Removed | AIC |
| RUF | 164.11 |
| Sex | 165.31 |
| Race | 165.45 |
| RWHAP | 165.88 |
| None | 167.3 |
| Age | 171.88 |

The second step in AIC-based model selection. Considers the newly updated model with five remaining variables and selects a variable to remove to obtain a lower AIC value.

**Supplemental Table A3: Final (Fifth) Step Function Measure of AIC Value**

| **Final (fifth) step (AIC = 158.87)**  suppression ~ age | |
| --- | --- |
| Variable Removed | AIC |
| None | 158.87 |
| Age | 164.35 |

The final step in AIC-based model selection. The model with the lowest AIC value was reached at the fifth iteration.

**Supplemental Table A4: Complete AIC Model**

| **Complete AIC Model** | | | | |
| --- | --- | --- | --- | --- |
| RUF | Categories for factors | Frontier | Rural | Urban |
| Age (years) | Mean (SD) | 50.7 (14.2) | 50.2 (11.7) | 52.4 (12.7) |
| RWHAP | no | 35 (34.3) | 19 (38.0) | 21 (17.5) |
|  | yes | 67 (65.7) | 31 (62.0) | 99 (82.5) |
| Insurance Status | no | 15 (14.7) | 5 (10.0) | 18 (15.0) |
|  | unknown | 22 (21.6) | 13 (26.0) | 10 (8.3) |
|  | yes | 65 (63.7) | 32 (64.0) | 92 (76.7) |
| Biological Sex | F | 21 (20.6) | 7 (14.0) | 18 (15.0) |
|  | M | 81 (79.4) | 43 (86.0) | 102 (85.0) |
| Race | non-white | 19 (18.6) | 4 (8.0) | 16 (13.3) |
|  | white | 83 (81.4) | 46 (92.0) | 104 (86.7) |
| Virally suppressed | no | 11 (10.8) | 3 (6.0) | 10 (8.3) |
|  | yes | 91 (89.2) | 47 (94.0) | 110 (91.7) |

The complete model was utilized, containing the six variables defined in the Data section.
